# Cost-Effectiveness of Point-of-Care A1C Tests in a Primary Care Setting

**DOI:** 10.3389/fphar.2020.588309

**Published:** 2021-01-19

**Authors:** Lorena de Sousa Rosa, Sóstenes Mistro, Marcio Galvão Oliveira, Clavdia Nickolaevna Kochergin, Mateus Lopes Cortes, Danielle Souto de Medeiros, Daniela Arruda Soares, José Andrade Louzado, Kelle Oliveira Silva, Vanessa Moraes Bezerra, Welma Wildes Amorim, Mark Barone, Luiz Carlos Passos

**Affiliations:** ^1^ Program of Post-Graduation in Medicine and Health, Federal University of Bahia, Salvador, Brazil; ^2^ Program of Post-Graduation in Collective Health, Multidisciplinary Institute of Health, Federal University of Bahia, Vitória da Conquista, Brazil; ^3^ Multidisciplinary Institute of Health, Federal University of Bahia, Vitória da Conquista, Brazil; ^4^ Departament of Natural Sciences, State University of Southwest Bahia, Vitória da Conquista, Brazil; ^5^ Intersectoral Forum to Fight NCDs in Brazil, São Paulo, Brazil

**Keywords:** low and middle-income countries, primary health care, point-of-care testing, diabetes mellitus, glycated hemoglobin A, cost-effectiveness

## Abstract

**Objective: **We evaluated the cost-effectiveness of the point-of-care A1c (POC-A1c) test device vs. the traditional laboratory dosage in a primary care setting for people living with type 2 diabetes.

**Materials and Methods: **The Markov model with a 10-year time horizon was based on data from the HealthRise project, in which a group of interventions was implemented to improve diabetes and hypertension control in the primary care network of the urban area of a Brazilian municipality. A POC-A1c device was provided to be used directly in a primary care unit, and for a period of 18 months, 288 patients were included in the point-of-care group, and 1,102 were included in the comparison group. Sensitivity analysis was performed via Monte Carlo simulation and tornado diagram.

**Results: **The results indicated that the POC-A1c device used in the primary care unit was a cost-effective alternative, which improved access to A1c tests and resulted in an increased rate of early control of blood glucose. In the 10-year period, POC-A1c group presented a mean cost of US$10,503.48 per patient and an effectiveness of 0.35 vs. US$9,992.35 and 0.09 for the traditional laboratory test, respectively. The incremental cost was US$511.13 and the incremental effectiveness was 0.26, resulting in an incremental cost-effectiveness ratio of 1,947.10. In Monte Carlo simulation, costs and effectiveness ranged between $9,663.20–$10,683.53 and 0.33–0.37 for POC-A1c test group, and $9,288.28–$10,413.99 and 0.08–0.10 for traditional laboratory test group, at 2.5 and 97.5 percentiles. The costs for nephropathy, retinopathy, and cardiovascular disease and the probability of being hospitalized due to diabetes presented the greatest impact on the model’s result.

**Conclusion: **This study showed that using POC-A1c devices in primary care settings is a cost-effective alternative for monitoring glycated hemoglobin A1c as a marker of blood glucose control in people living with type 2 diabetes. According to our model, the use of POC-A1c device in a healthcare unit increased the early control of type 2 diabetes and, consequently, reduced the costs of diabetes-related outcomes, in comparison with a centralized laboratory test.

## Introduction

People living with diabetes mellitus (DM) have an increased risk of disabilities and early death due to macro and microvascular complications resulting from poor glycemic control (Camargos et al., 2018). Achieving glycemic targets in DM is directly associated with the appropriate use of medicines, changes in lifestyle, and the monitoring of blood glucose levels through a glucometer and periodic glycated hemoglobin A1c tests (Karkare et al., 2019). The A1c test allows the healthcare team to determine which individuals need to have their treatment reviewed, with the aim of avoiding both overtreatment and the worsening of their clinical presentation due to the lack of glycemic control (Hirst et al., 2017). Moreover, A1c tests predict which individuals have a higher risk of complications due to their target status (Camargos et al., 2018). However, there are several obstacles that prevent people living with DM from having A1c tests regularly. For example, people with low-income and rural populations face this difficulty (Zheng et al., 2018).

In 2019, 463 million people aged 20 to 79 were living with type 2 diabetes around the world, and almost 80% belonged to low- and middle-income countries (LMIC). The predicted mortality rate was 11% (International Diabetes Federation, 2019). Besides the alarming mortality numbers, DM also represents an important source of healthcare-related expenditure. A person living with DM represents yearly average direct costs estimated at US$11,804.7, which include expenses for emergency visits, outpatient clinic visits, and hospitalizations at general wards or intensive care units (Wong et al., 2018). Hospitalization may cost, on average, US$2,127.10 per patient per admission (Li et al., 2019). Thus, the high prevalence of type 2 diabetes and the high cost of treating the related complications ascribe a huge economic burden on health systems worldwide, ranging from US$1,000 per capita annually in low-income countries to more than US$10,000 for high-income countries (Seuring et al., 2015). Not surprisingly, these costs increased considerably if there were comorbidities or complications leading to hospitalizations (Chen et al., 2017).

Individuals with poor glycemic control have accelerated the progression of diabetic retinopathy (Osataphan et al., 2017). According to the Diabetes Complication Severity Index, within 10 years of non-glycemic control, patients with A1c over 8% have a 16% greater risk of developing micro and macrovascular complications, such as cardiovascular disease and nephropathy, and a higher mortality risk (Pantalone et al., 2018). Controlling blood glucose levels is crucial to reducing costs and improving the quality of life of people living with DM. Some strategies optimize control, such as multidisciplinary protocols (Henriques et al., 2018), new methods of insulin administration (Roze et al., 2019), new drugs (Durden et al., 2016), telemonitoring of patients (Warren et al., 2018), weight loss and exercising (Karkare et al., 2019), and increasing the number of medical consultations (Schwab et al., 2016). Despite these efforts, without the A1c test, it is difficult to timely identify individuals who are out of their glycemic target and adjust their therapy, which would prevent the advancement of vascular lesions, hospitalizations, and early death. However, the cost of an A1c test, along with travel to a centralized laboratory, collecting a blood sample, and returning later for the test result, before the medical appointment in primary care, may lead to failures in individual follow-up (Alonso-Fernández et al., 2015).

The increased reliability of point-of-care (POC) devices for A1c testing has been shown to improve individual monitoring of blood glucose levels, because they can be used directly at primary care units (PCU), just before visiting the physician. With immediate access to the A1c test result, in many cases, changes in therapy can be made promptly to quickly improve glycemic control. Moreover, using POC devices at PCU could probably increase access to A1c tests for underserved and rural populations living with DM. However, POC devices and cartridges for A1c tests are expensive, which may be an obstacle for widespread use. The aim of this study was to evaluate the cost-effectiveness of a POC device for A1c dosage vs. traditional laboratory dosage in a primary care setting for people living with type 2 diabetes.

## Materials and Methods–Study Design

We developed a Markov-based economic model to evaluate the cost-effectiveness of POC-A1c for the municipal government perspective, for routine monitoring of people living with type 2 diabetes. Our main assumptions are: 1) Improved control of glycemic levels results in risk reduction of diabetes-related complications (Huang et al., 2011); and 2) A1c control directly reflects glycemic control (Yazdanpanah et al., 2017).

### Funding and Organization of Primary Care in Brazil

In Brazil, primary care is part of the public Unified Health System (SUS) funded by the federal government, states, and municipalities. The resources are managed by municipalities, which are responsible for local health policies and providing services. Hospitalizations in municipal or state hospitals caused by DM or hypertension are funded by municipalities. Each healthcare team in a PCU comprises a physician, nurse, dentist, nurse technician, and a group of community health workers, who are responsible for 2,000–3,500 people (generally, this number is higher). DM and hypertension management is managed mainly by urban and rural PCUs. At the local PCU where this study was conducted, A1c tests are conducted in a central laboratory after being requested by physicians. The collection of blood samples requires that people living with DM travel to the laboratory. As the demand for appointments at the PCU and the demand for laboratory tests is high, even when all goes well, the results may take 8–12 weeks to reach the physician, which could keep the individual out of their glycemic target for a longer period than desired (Brasil, 2017).

### Data Sources for the Economic Model

This study evaluated data from people living with DM who were seen at the 16 PCUs in the urban zone of Vitória da Conquista. Located in the northeast region of Brazil, this city has 338,480 inhabitants (Prefeitura Municipal de Vitória da Conquista, 2019) and a human development index of 0.708 (IBGE, 2017). The 18-month follow-up of participants was conducted by a research group as part of the HealthRise Program, which is a global initiative aimed at improving both access and quality care for individuals in underserved communities with DM and hypertension. The local project included support for workflow reorganization, purchasing medical and computing devices, implementing electronic medical records, training healthcare providers in protocols for DM and hypertension management, qualifying community health workers, conducting health fairs to detect target or undiagnosed people living with DM and hypertension, and monitoring the results of clinical test data such as A1c and blood pressure. Additionally, some new technologies, such as POC-A1c devices, were assessed in a real-life setting of primary care. A POC-A1c device was allocated to one PCU for 6 months. At that unit, individuals without recent A1c test results were tested prior to the physician’s appointment. Individuals who presented an A1c test result above the target level were scheduled for a new test 3 months later, in accordance with the routine PCU workflow. Informed consent was required from all individuals. No direct physician-patient intervention was made. The work that the physicians did in relation to their patients was not interfered with. Unitary costs were US$3,976.35 for the Roche Cobas b 101^®^ POC device and US$8.48 for the cartridge.

## Literature Review

Data related to costs and the probabilities of controlling diabetes, developing complications, and death were searched in PubMed^®^ and Science Direct^®^. The mesh terms and keywords used were “type 2 diabetes,” “complications,” “Brazil,” “mortality,” “control rate,” “costs,” and “comorbidities”. They were mixed in several different combinations during the search. The search was filtered by title, and no time period was selected. When Brazilian data were not found for the probabilities, data were extracted from papers published for LMIC.

The costs and probabilities of each evaluated complication extracted from the literature are available in Table 1. The complications considered were cardiovascular disease (CVD), diabetic foot, retinopathy, nephropathy, and hospitalization. Most of the costs that are used refer to the reality in Brazil, which makes the model closer to an accurate result. However, few studies have researched the probabilities of these selected complications in Brazil. For diabetic foot, the mean cost of three complications applied in the model was US$166.27. The price for the A1c conventional laboratory test was extracted from national databases (US$2.65). Costs which were available in different currencies were converted using the Purchasing power parity criteria based on the statistics from the World Bank.

**TABLE 1:** Costs and probabilities of type 2 diabetes-related complications used in the economic model.

Complications	Value/patient/year (US$)	Source of data (Ref.)
Costs		
Cardiovascular disease	1,529.00	Rosa et al. (2018)
Retinopathy	621.00	Bahia et al. (2019)
Nephropathy	1,602.00	Bahia et al. (2019)
Hospitalization	3,917.00	Henriques et al. (2018)
Diabetic foot with healing^a^	162.10	Rezende et al. (2008)
Diabetic foot with minor amputation^a^	112.90	Rezende et al. (2008)
Diabetic food with major amputation^a^	223.80	Rezende et al. (2008)
Diabetes general cost	1,844.00	Borges et al. (2014)
Probability of occurrence		
Cardiovascular disease	0.129	Santos et al. (2015)
Retinopathy	0.1340	Henriques et al. (2018)
Nephropathy	0.1680	Henriques et al. (2018)
Hospitalization	0.1895	Li et al. (2018)
Diabetic foot	0.0310	Bao et al. (2017)
Probability of death		
Cardiovascular disease	0.2840	Bujang et al. (2018)
Retinopathy	0.0000	Schmidt et al. (2015)
Nephropathy	0.0381	Sinkeler et al. (2013)
Hospitalization	0.0617	Li et al. (2018)
Diabetic foot	0.0740	Vassallo et al. (2019)

^a^ Values converted to USD according to the currency exchange on 07/26/2019 and updated to 2020 values, according to the World Bank annual deflator data.

### Markov Model

**FIGURE 1:**
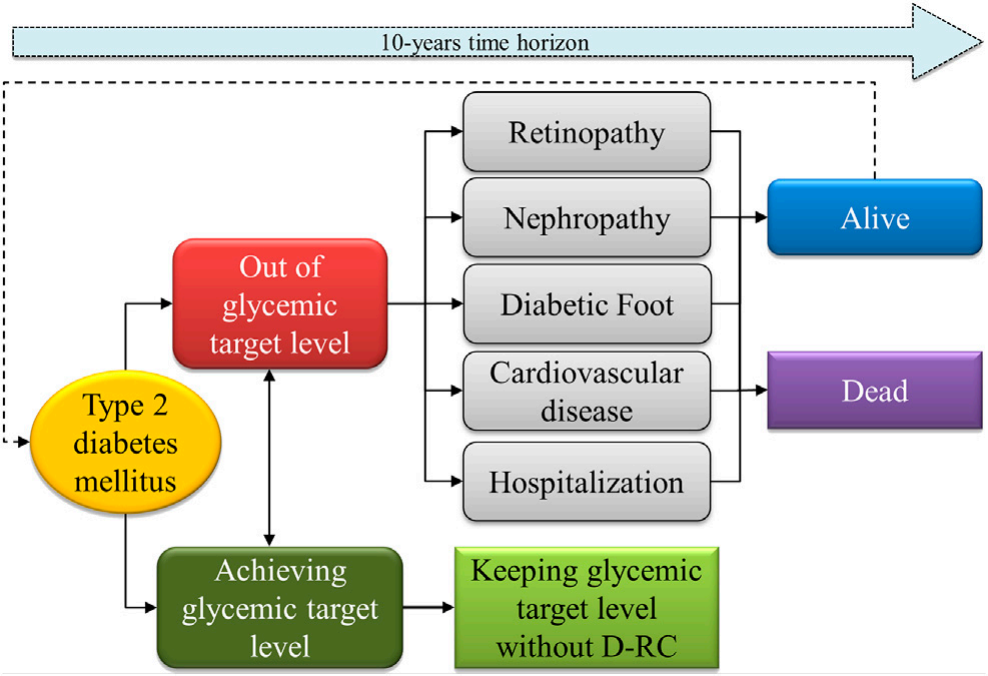
Schematic flowchart of the Markov model used to assess Cost-effectiveness of the POC vs. traditional A1c tests in a primary care setting.

A transitional Markov model was built to compare the cost-effectiveness of the POC-A1c device vs. traditional laboratory A1c testing (High Performance Liquid Chromatography–HPLC) method in a centralized municipal laboratory, considering the progression of a person with type 2 diabetes over a time horizon of 10 years. The structure of the economic model is detailed in Figure 1. Probabilities for transition states (complications) were extracted from the literature review. The control rate for the POC device group of A1c tests was extracted from the HealthRise dataset, corresponding to 0.14, while the control rate for the traditional laboratory test was 0.0738, according to previous literature (Brown et al., 2017). The effectiveness for both groups were extracted from the cohort as 0.0335 for the POC device group and 0.3 for the traditional laboratory test. Each cycle of the Markov model was set at 3 months, according to the recommended A1c reassessment time frame. Effectiveness was defined as achieving target levels after a 6-month period. The target level was defined as an A1c of 7.5%. A discount rate of 4% per year was applied to costs and outcomes. A half-cycle correction was performed to reduce the bias of the model. It was assumed that all individuals entered the cohort out of the glycemic target level.

### Sensitivity Analysis

A tornado diagram was drawn to understand the influence of each model input parameter. The probabilistic sensitivity analysis by the Monte Carlo simulation was conducted to check model reliability. The economic model and sensitivity analysis were performed using TreeAge Pro 2020 - R1.2 (TreeAge Software Inc., MA, United States).

## Results

For 18 months, the local HealthRise team monitored the records of 1,390 individuals with DM. Of these, 288 (20.7%) patients were seen in the PCU where the POC-A1c device was available, and 1,102 (79.3%) were seen in 15 other PCUs. Baseline characteristics of the individuals included in the POC-A1c device group vs. the traditional laboratory test group were, respectively, as follows: 1) individuals with results for A1c available [219 (76%) vs. 397 (36%), *p* < 0.0001]; 2) sex [81 (37%) male and 138 (63%) female vs. 91 (23.4%) and 304 (76.6%), *p* < 0.001]; 3) mean age in years (standard deviation) [61.9 (0.91) vs. 56.8 (0.55), *p* < 0.001]; 4) hypertension as comorbidity [201 (91.7%) vs. 371 (93.5%), *p* = 0.544]; 5) A1c median (interquartile range) [7.8% (6.7%–9.5%) vs. 7.9% (6.8%–10.6%), *p* = 0.025]; and 6) number of medical appointments–mean (standard deviation) [1.19 (0.05) vs. 1.42 (0.06), *p* = 0.005]. The endline, as the effectiveness of the A1c target is achieved, was 0.14.

In the cost-effectiveness analysis, no dominance was observed between the two strategies. POC-A1c presented a mean cost of US$10,503.48 per individual and an effectiveness of 0.35, vs. US$9,992.35 and 0.09, respectively, for the traditional laboratory test in the 10-year time horizon. Consequently, POC-A1c device presented an incremental cost of US$511,13 for an incremental effectiveness of 0.26, resulting in an incremental cost-effectiveness ratio of US$1,947.10.

**FIGURE 2:**
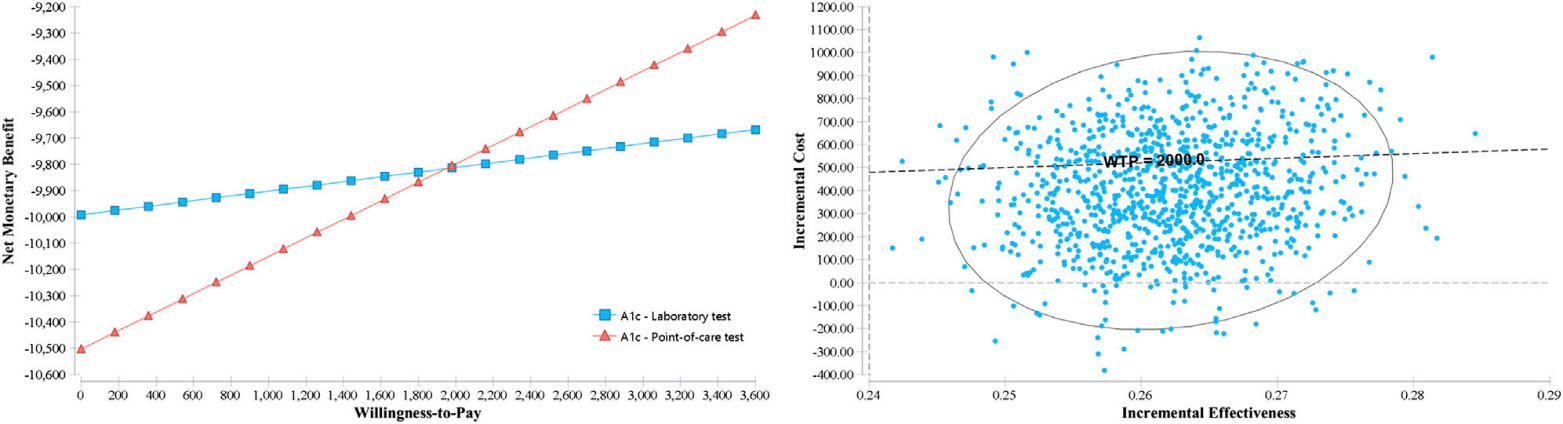
Probabilistic sensitivity analysis by Monte Carlo simulation with the variation of the Net Monetary Benefit vs. Willingness-to-Pay **(left)** and incremental cost-effectiveness scatter plot **(right)**.

**FIGURE 3:**
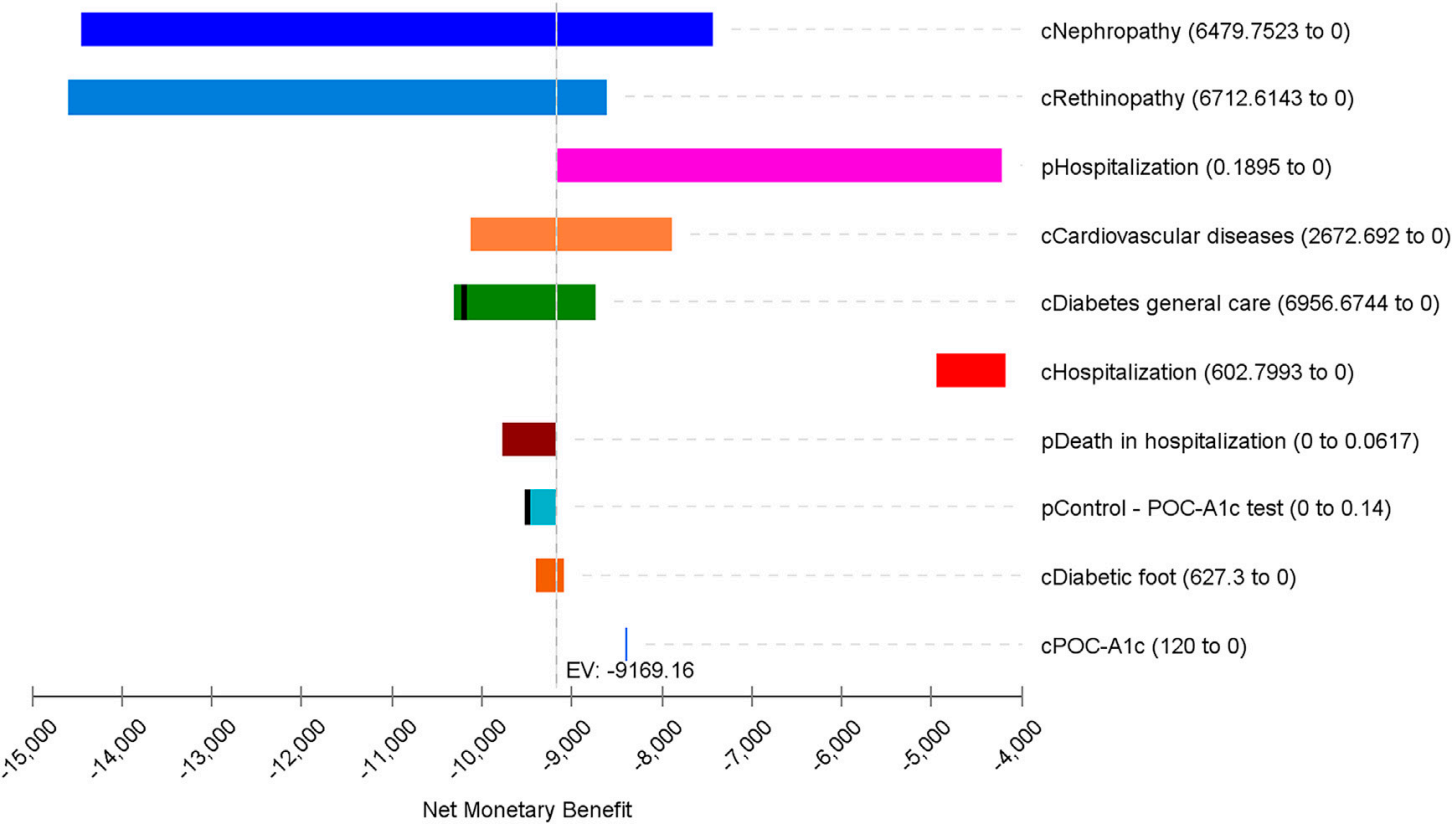
One-way sensitivity analysis by a tornado diagram ranking variables by the impact of the results on the economic model with a Willingness-to-Pay threshold of US$2,000.00.

In the sensitivity analysis by Monte Carlo simulation with 1,000 random trials, the net monetary benefit (NMB) reached by the POC-A1c test overcomes the traditional laboratory test at a willingness-to-pay (WTP) value of US$2,000 per person in the 10-year period (Figure 2). The same WTP threshold applied in the scatter plot (Figure 2) shows how each iteration between incremental effectiveness and incremental cost happened in the model. The tornado diagram (Figure 3) shows the main variables affecting the results of the economic model. The cost of nephropathy, retinopathy, and CVD, and the probability of hospitalization due to diabetes-related complications had the greatest impact on the cost-effectiveness of the comparators.

## Discussion

Despite being more expensive than the laboratory method, we found in our setting that the POC-A1c device is an equivalent alternative for monitoring the blood glucose levels of patients with type 2 diabetes. The POC-A1c device is faster in providing results compared to the traditional laboratory test. When the device is available at the PCU, more individuals can be tested prior to an appointment with a physician. The results indicate that, for a 10-year period, the total cost of caring for people living with type 2 diabetes is slightly higher if A1c tests were performed by the POC-A1c device. These data suggest that timely access to the exam, observed by inserting POC-A1c in primary care routine, may lead to the faster achievement of the desired A1c target, potentially minimizing diabetes-related complications, which result in health, economic, and social burdens.

The periodic monitoring of A1c directly affects decisions regarding possible changes in medication, diet, alternative therapies, and assessment adherence, which should promptly be implemented if out-of-target results are obtained (Nerat et al., 2016; Laiteerapong et al., 2018). However, a lack of the monitoring test leaves professionals and individuals without a control assessment parameter, which delays the achievement of treatment goals. This time loss harms the quality of life, increases health and social costs, and exacerbates early deaths. This study demonstrates that providing tests directly in PCU by using POC-A1c devices may expand access for proper monitoring of DM, especially for underserved populations assisted by the public healthcare system. These individuals encounter a stressful treatment process due to the high frequency of travel between the PCU and the laboratory.

Previous studies have examined the use of POC-A1c devices in hospitals (Patzer et al., 2018) or for early diagnosis in tertiary care (Gomez-Peralta et al., 2016). It was found to improve blood glucose levels in primary care (Motta et al., 2017). Although POC-A1c devices are convenient and reliable for diabetes management (Grant et al., 2017), the costs of the devices and the supplies for A1c tests discourage their widespread use, and these costs are accounted for only at the time of purchase. This study found that the costs of purchase are offset by savings from decreased hospitalizations, heart attacks, strokes, amputations, ophthalmic procedures, blindness, dialysis, or other DM-related complications.

The sensitivity analysis indicates that the cost of general care, the POC-A1c device, and DM-related complications impacted the results of the model by increasing costs, while the probability of DM control observed in the POC device group was the main factor that led to lower costs. The cost of the POC-A1c device is probably the most sensitive factor, depending on the device manufacturer, the number of devices, and test cartridges. Thus, purchasing more devices and cartridges could reduce prices and increase the cost-effectiveness of the POC-A1c device. Large municipalities, states, or the ministry of health may be the only parties capable of purchasing multiple devices. Therefore, the results may not be applicable to smaller settings. Additionally, if laboratory tests are more easily accessible than in this study, or if the costs of diabetes care and the rates of complications are lower than those in this study, then these results have to be applied with caution.

This study has some limitations. First, the model does not consider the costs paid by the individuals to travel to the centralized laboratory for testing, receiving results, and having a follow-up consultation. This oversight may impact the results and is a possible avenue for future research. Second, the study uses observational and secondary data rather than a clinical trial to test effectiveness. However, by assessing the POC-A1c device as close as possible to an actual scenario, we believe that our results can sufficiently provide a snapshot of what really happens if physicians receive A1c test results on time and are, therefore, able to detect out-of-target individuals and promptly review their treatment plan. Using a clinical trial to test cost-effectiveness is a possible avenue for future research. Other difficulties that could possibly be faced in a real-life scenario include the distance from home to laboratories, the overload of primary healthcare, the shortage of antidiabetic drugs, limited therapeutic arsenal (unavailability of new drugs), individual non-adherence, and economic conditions restraining healthy habits. Third, different data sources were used to define probabilities and costs in transition states of DM-related complications, with an approximation to LMIC. Finally, some costs included in the modeling refer only to hospitalization due to diabetes-related complications, because wider data were not available. However, we believe this approach covers a substantial part of the costs assumed by the municipal governments in our setting, in addition to the costs of the local primary care system.

The adoption of the POC for glycemic control through A1c measurement helps overcome clinical inertia, as it leads to the earlier-than-expected use of oral antidiabetic drugs or insulin. Additionally, this adoption can lead to the start of new healthcare policies for managing type 2 diabetes in the public health system, emphasizing individualized follow-up, education, and empowerment tactics to change behaviors and improve lifestyles. Clinical inertia increases the risk of comorbidities and mortality due to diabetes, especially for patients with poor glycemic control, which increases the costs associated with type 2 diabetes. Increased delays in getting the right medications would decrease the prognoses of people living with diabetes (Reach et al., 2017; Ali et al., 2020).

## Conclusion

This study showed that using POC-A1c devices in primary care settings is a cost-effective alternative for monitoring glycated hemoglobin A1c as a marker of blood glucose control in people living with type 2 diabetes. Compared to a centralized laboratory test, the use of the POC-A1c device in a healthcare unit increased the chance of the early control of type 2 diabetes and reduced costs in relation to DM-related outcomes.

## Data Availability Statement

The raw data supporting the conclusions of this article will be made available by the authors, without undue reservation.

## Ethics Statement

The studies involving human participants were reviewed and approved by Comitê de Ética em Pesquisa do Instituto Multidisciplinar em Saúde da Universidade Federal da Bahia, Vitória da Conquista, Bahia, Brazil. The patients/participants provided their written informed consent to participate in this study.

## Author Contributions

SM, MO, and LR contributed to the study conception and design. Data acquisition, analysis and interpretation, and the manuscript draft were performed by SM and LR. MB, CK, KS, MC, DM, DS, JL, VB, WA, and LP actively contributed to the critical revision of the manuscript.

## Funding

The HealthRise program is a global initiative funded by the Medtronic Foundation (USA) in collaboration with the Institute for Health Metrics and Evaluation (IHME) and ABT Associates. Institutional Development Support Program of the Unified Health System ‐ SUS (PROADI)/Ministry of Health.

## Conflict of Interest

The authors declare that the research was conducted in the absence of any commercial or financial relationships that could be construed as a potential conflict of interest.
